# A Potential Link Between Prolonged Cork Exposure and Intestinal-Type Sinonasal Adenocarcinoma – Special Findings of a Retrospective Cohort Analysis

**DOI:** 10.3389/fonc.2020.565036

**Published:** 2020-09-18

**Authors:** Diogo Alpuim Costa, Ana Monteiro, Teresa André, Susana Esteves, Isabel Sargento, Margarida Ferreira, Teresa Alexandre, Ana Clara, João Freire, António Moreira

**Affiliations:** ^1^Department of Haematology and Oncology, CUF Oncologia, Lisbon, Portugal; ^2^Department of Medical Oncology, Instituto Português de Oncologia de Lisboa Francisco Gentil, E.P.E., Lisbon, Portugal; ^3^Department of Medical Oncology, Hospital Central do Funchal, Madeira, Portugal; ^4^Department of Clinical Trials, Instituto Português de Oncologia de Lisboa Francisco Gentil, E.P.E., Lisbon, Portugal

**Keywords:** sinonasal cancer, sinonasal adenocarcinoma, intestinal-type sinonasal adenocarcinoma, cork, wood dust, exposure, occupational cancer

## Abstract

**Introduction:**

intestinal-type sinonasal adenocarcinoma (ITAC) is a rare epithelium tumor of the nasal cavities and paranasal sinuses. Exposure to wood and leather dusts is a strong etiological factor related to its development. Prolonged cork exposure has rarely been associated.

**Materials and Methods:**

thirty-seven-year (1981–2018) retrospective cohort analysis of all consecutive patients with sinonasal cancer (SNC) followed at our institution. Medical records were reviewed to determine patient demographics, occupational/environmental exposure, location and extent of the tumor, stage, histopathology findings, treatment strategies, and oncologic outcomes. Survival analysis was done using Kaplan–Meier method.

**Results:**

we evaluated 379 patients with SNC, including 39 (10.3%) ITAC. Patient median age was 73 years (range 49–87), 56% male and 69% with identified professional occupational exposure (54% for cork; 69.2% considering only those for which an agent has been identified). Seventy-two percent had locally advanced disease (stage III or IVA–B). The initial treatment was surgery in 77%, and 54% received adjuvant radiotherapy. The median time to progression, progression-free survival, and overall-survival was 2.36 years (95% CI 1.54–8.70), 1.96 years (95% CI 1.43–3.74), and 3.51 years (95% CI 2.33–10.02), respectively.

**Conclusion:**

ITAC is an uncommon malignancy that grows silently, which contributes to delayed diagnosis, advanced stage and low survival rates. In our cohort, we observed a high prevalence of cork occupational exposure. This finding may lead to the implementation of protection measures and suggest a potential link to be further studied.

## Introduction

Sinonasal cancer (SNC) is relatively uncommon in the general population, accounting for 0.4% of all human neoplasms and representing 3–5% of all head and neck cancers, with an annual European incidence of approximately 1 case per 100,000 inhabitants (1, 2). Over three-quarters of all SNC consist of epithelial tumors, among which the most common histology types are sinonasal squamous cell carcinoma (SNSCC) (50–80%) and sinonasal adenocarcinoma (SNAC) (10–20%), although these proportions vary geographically due to several factors (1).

Sinonasal adenocarcinoma can originate in the respiratory epithelium or the underlying mucoserous glands. According to the World Health Organization (WHO) histological classification, there are two main types of non-salivary-gland-type adenocarcinoma: intestinal-type adenocarcinoma (ITAC) and non-intestinal-type adenocarcinoma (NITAC) (3). ITAC is more common than NITAC, with an age-standardized European (4) incidence per 100,000 inhabitants-years of 0.26 cases in men and 0.04 in women, whereas in the United States (5), these values are 0.058 in men and 0.034 in women. In Europe, there are regions with higher incidence such as the northern part of Spain (6) with 0.19 cases per 100,000 inhabitants-year or Denmark (7) that reported the highest rates, with incidence continuing to increase, in contrast to trends in other countries which either remained relatively stable, or were decreasing slightly.

Intestinal-type adenocarcinoma is morphologically indistinguishable from gastrointestinal tract adenocarcinoma. It may occasionally resemble colorectal tubular adenoma and even non-neoplastic intestinal epithelium (8, 9). On the other hand, less is understood about NITAC, which consist of adenocarcinoma with distinct immunophenotype that lacks the aforementioned intestinal morphology and is subclassified as low-and high-grade tumors (10).

Furthermore, the classifications by Barnes (11) and, Kleinsasser and Schroeder (12) distinguish five histopathological types of ITAC that are associated with clinically significant differences in outcomes: papillary tubular cylinder cell I (PTCC-I), colonic (PTCC-II), solid (PTCC-III), mucinous, and mixed or transitional. The most frequent type is colonic (40%), followed by solid (20%), papillary (18%), the mucinous and mixed varieties (both 22%) (1, 11).

Intestinal-type adenocarcinoma is more frequent in the ethmoidal sinus (40%), followed by nasal cavity (27%) and maxillary sinus (20%), and exceptionally arises in other sites of the nasal cavity. Median age at diagnosis is 64–68 years (13), with a peak of incidence at 60–69 years in the United States (5) and 75–84 years in Italy (4). There is a strong male predominance (up to sixfold more commonly than females), reflecting occupational exposure to several industrial compounds (1, 14).

Intestinal-type adenocarcinoma usually grows silently with non-specific symptoms, which leads to late diagnosis and low survival rates. Primary symptoms include (unilateral) nasal obstruction, rhinorrhea, epistaxis, local pain, ocular symptoms (with proptosis, diplopia, and visual loss), headache and nausea due to intracranial growth, or presence of an ulcerated mass with facial deformation due to bone invasion. Nodes and distant metastases are uncommon at presentation, perhaps because the pattern of tumor spread is largely dependent on the sinus origin and lymphatic drainage. Patient survival depends on local control that could be challenging attending to the anatomical proximity of the orbit and brain, and local recurrence is frequent (1, 2, 6, 8, 13).

The nasal cavity is the most common portal of entry and a well-known target site for a wide range of pollutants and chemically induced carcinogenicity. Occupational risk factors that have been related to a higher incidence of SNC include chronic exposure to wood and leather dust, nickel and chromium compounds, formaldehyde and other solvents, and polycyclic aromatic hydrocarbons. Occupational epidemiological studies have also shown an increased risk in workers employed in leather tanning, production of isopropyl acid, welders, and with cork products. Cigarette smoking and heavy alcohol consumption have long been known to increase the risk of head and neck malignancies, but no significant association has been shown with SNC. Other established intrinsic or acquired risk factors such as previous radiotherapy, inverted papilloma (related to human papillomavirus type 16 and 18) or chromosomal and genetically determined alterations could be responsible for a high risk of SNSCC (1, 4, 6, 12, 15–21).

The association of ITAC and wood/leather dusts is already established, with variations worldwide that may be related to unidentified genetic susceptibility factors or exposure to different types of wood or other compounds (outside of Europe, only 20% of patients with ITAC have a history of exposure to wood) (1).

However, there is little evidence in the literature regarding the association between prolonged cork exposure and ITAC (18–20).

Due to the developed cork industry in Portugal and to the scarce ITAC cancer reports related to its exposure, this study aimed to:

• To describe the epidemiological, clinical, pathological, and prognostic characteristics of a series of consecutive cases of ITAC;
• To report the potential occupational risk factors in this cohort;
• To characterize our experience and outcomes in the management of patients with ITAC.

## Materials and Methods

### Study Design and Data Collection and Selection of Patients

We performed a retrospective cohort analysis of all consecutive patients with histologically confirmed primary SNC, diagnosed from 1981 to 2018 and treated at the Instituto Português de Oncologia de Lisboa Francisco Gentil, E.P.E. (IPOL-FG), a tertiary comprehensive cancer care center that is a reference for the treatment of head and neck cancer. Cases were identified from the Cancer Registry South of Portugal (ROR-Sul) and comprised diagnoses up to December 31, 2018. Cut-off date for follow-up was October 2019.

Data concerning demographics, location and extent of the tumor, stage, histopathology findings, treatment strategies, and oncologic outcomes were retrieved from medical records.

All patients were retrospectively evaluated for occupational exposure and extraprofessional risk factors such as smoking habits, through clinical process consultation, or by face-to-face questionnaire or an authorized phone call. In cases of death or loss for follow-up, supplementary information was provided by the national health system databases and/or by family or patients’ acquaintances. Whenever a potential occupational hazard was identified, we performed a detailed systematic characterization of exposure to wood dust and cork.

The tumors were re-staged according to the American Joint Committee on Cancer (AJCC) TNM staging, 8th Edition, 2017. Treatment strategies included surgery, radiotherapy, and chemotherapy separately or in combination.

### Statistical Analysis

Descriptive analysis of clinical, epidemiological, and demographic data was done using absolute and relative frequencies for categorical variables and median, minimum, and maximum for quantitative variables. The outcomes time to progression (TTP), progression-free survival (PFS), and overall-survival (OS) were calculated using Kaplan–Meier method. TTP was defined as time from start of first treatment to progression or recurrence (patients that died without recurrence were censored at the date of last follow-up), PFS as time from the start of first treatment until progression, and OS as time from diagnosis until death from any cause. Statistical analysis was done using R^1^.

## Results

From 379 patients with SNC, 39 (10.3%) were ITAC. At diagnosis, the median patient age was 73 years (range 49–87), of whom the majority 22 (56%) were men. Any occupational exposure was found in 27 patients (69%), including 21 (54%) with a history of professional exposure to cork. Among patients with a history of occupational exposure to an agent, it is noted that 69.2% had worked in the processing of cork. Other workers were from long-term exposure to hardwood dust (*n* = 3; 8%), textile industry, wood stove cooks, electromechanical, and terracotta insulation factory workers. The median duration of exposure was 30 years (range 10–51) in cork workers. Twelve patients (31%) had smoking habits, and 18% had alcohol consumption (Table 1).

**TABLE 1:** Clinical and demographic characteristics at diagnosis and exposure information.

Variable	*N* (%)
**Gender**	
Male	22 (56.41)
Female	17 (43.59)
**Age, years**	
Median (min–max)	73 (range 49–87)
**Occupational exposure**	
Yes	27 (69.23)
No	12 (30.77)
Agent	*N* (%); (median) duration of exposure (years)
Cork	21 (53.85); 30 years (range 10–51)
Wood dust	3 (7.69); 50 years, NA
Coal	1 (2.56); 10 years
Isomastic; terrace insulators	1 (2.56); 5 years
Textile industry	1 (2.56); 20 years
**Disease stage (AJCC TNM staging)**	
I	2 (5.13)
II	9 (23.07)
III	14 (35.90)
IVA	6 (15.38)
IVB	8 (20.51)
**ITAC histologic subtype**	
ITAC NOS	4 (10.26)
ITAC colonic	23 (58.98)
ITAC mucinous	9 (23.07)
ITAC papillary	3 (7.69)
**Year of diagnosis (1981–2018) (5-year interval)**	
1981–1985	0
1986–1990	1 (2.56)
1991–1995	0
1996–2000	8 (20.51)
2001–2005	18 (46.16)
2006–2010	4 (10.26)
2011–2015	3 (7.69)
2016–2018*	5 (12.82)

AJCC, American Joint Committee on Cancer; ITAC, intestinal-type adenocarcinoma; NA, not available; NOS, not specified. *3 year interval.

Primary sites at diagnosis were: nasal cavity 26% (*n* = 10), ethmoid sinus 8% (*n* = 3); 62% (*n* = 24) involving multiple locations. Of the 21 patients exposed to cork dust, 14 had a disease with more than one location, including seven in the maxillary sinus. Nasal obstruction (79%), epistaxis (56%) were the most common presenting symptoms. Other symptoms included headache 15%, rhinorrhea 7.5%, and diplopia 5%. At the diagnosis time, the majority had locally advanced disease with stage III (*n* = 14, 36%) and stage IV (*n* = 14, IVA 15% and IVB 21%). Eleven patients (28%) presented with stage I/II. The ITAC was the most common type of adenocarcinoma, mainly colonic (*n* = 23, 59%), followed by mucinous (*n* = 9, 23%), and papillary (*n* = 3, 8%). The median time between the first symptom and the definitive diagnosis was 4 months (range 0–36) and from the diagnosis to the initial treatment was 2 months (range 0–8). Surgery was the primary form of treatment in 77% of patients. Adjuvant radiation was performed in 54% (Table 1).

**FIGURE 1:**
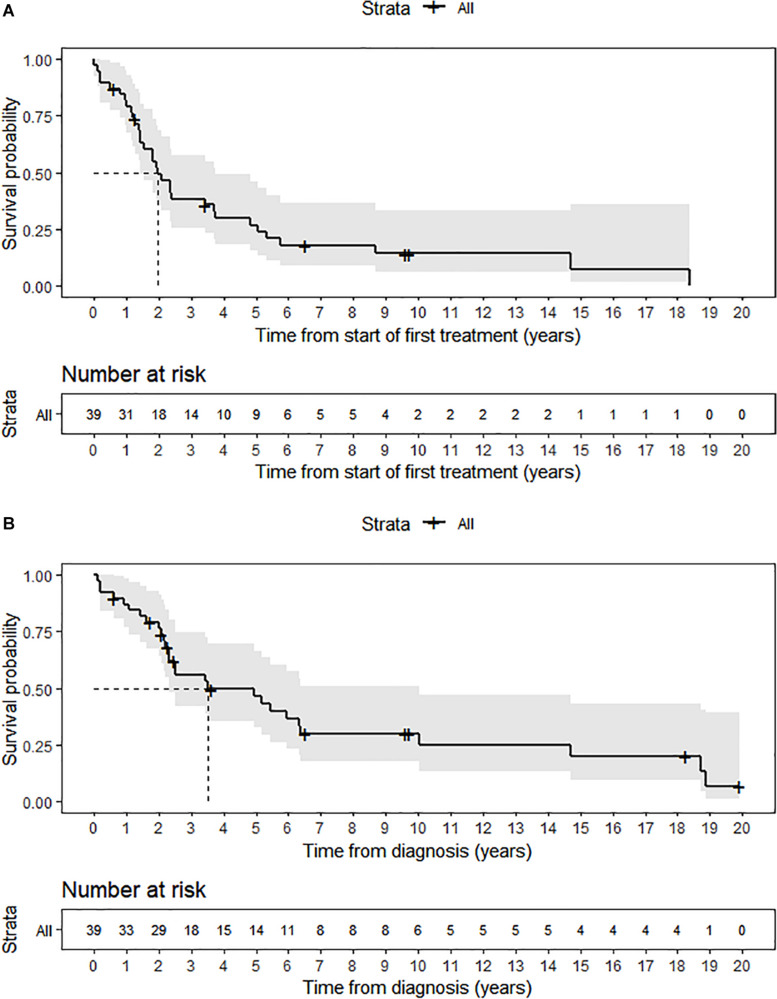
**(A)** Progression-free survival. **(B)** Overall-survival.

The median follow-up in patients alive was 3.6 years (range 0.63–19.9). We observed 24 progression or recurrence events and 28 deaths from any cause. The median TTP and PFS was 2.36 years (95% CI 1.54–8.70) and 1.96 years (95% CI 1.43–3.74), respectively. Moreover, the median OS was 3.51 years (95% CI 2.33–10.02) and 5-year. OS (5-OS) rate was 46.4% (Figures 1A,B). Of the patients with prolonged exposure to cork, only 7 (33.3%) were alive at the end of 5 years after the diagnosis of ITAC.

Between patients with stage I–II and stage III–IV a statistically significant difference in all the evaluated outcomes was documented: TTP (median 5.07 vs. 1.43; *p* = 0.023), PFS (median 4.82 vs. 1.43; *p* = 0.0328), and OS (median 10.02 vs. 2.52; *p* = 0.009).

## Discussion

In this retrospective study, we found some similar demographic and clinical characteristics to the data already published in literature (1, 8) – the male predominance, advanced age, clinical course, and chronic occupational exposure to some agents.

Interestingly, the men-to-women ratio was not what would be predictable for this type of cancer (up to sixfold), when compared to other European countries such as Italy (4). In fact, the ratio was similar to that observed in the United States (5). This fact is probably more related to the small sample size. However, it would be relevant to determine whether there are genetic polymorphisms that determine higher susceptibility to occupational agents (and in this case to cork) for the development of ITAC.

The primary site at diagnosis was nasal cavity, unlike what is described as being more frequent in the ethmoid sinus (although more than half of cases with multiple locations due to local invasion). According to Leivo (9), only exceptionally the tumor arises in the maxillary sinus (<10%), and in these cases are usually not related to wood dust exposure. However, of the total number of patients exposed to cork dust, two thirds had a disease with more than one affected site, and one third involved the maxillary sinus. This finding is associated only to the extent of the local disease and/or to a more specific pathophysiology related to exposure to cork.

The prognosis of ITAC is poor, with a 5-OS between 20 and 50% (21). In our series, the 5-OS rate and the considerable difference in all clinical outcomes between advanced and early-stage disease and a high overall percentage of local progression or recurrence were in line with prior studies. ITAC associated with wood dust exposure has a somewhat better prognosis (with 50% survival rates at 5 years) than sporadic ITAC (with 20–40% survival rates at 5 years) (21, 22). Although, in our subgroup of patients with prolonged exposure to cork, we did not find this confirmation, with only one third alive after 5 years of diagnosis. It is again related to the sample size and/or to a more aggressive biology associated with this more specific epidemiological factor. Well-differentiated papillary and colonic ITACs pursue a more indolent course, but solid and mucinous subtypes have poorer outcomes (21). We did not corroborate the previously reported tendency of the mucinous histology to a dismal prognosis (23).

Surgical resection combined with radiotherapy remains the gold-standard treatment of choice for most patients with ITAC (21). In this study, more than two thirds and more than half of the patients underwent surgery and adjuvant radiotherapy, respectively. Of note is the absence of systemic anti-neoplastic treatment, as this type of cancer is usually treated with local therapies.

The association between SNAC (mainly ITAC) and wood dust exposure was first described in 1923 by Moure and Portman (24). This data was confirmed in a study published in 1965 by Macbeth (25). Wood dust was identified as being carcinogenic in 1995 by the International Agency for Research on Cancer (IARC) (17), and in 1999 by a directive of the European Union (EU) (Directive 1999/38/EC of April 29, 1999) (26). NITAC is not related with wood dust and behaves differently (20). Although the actual carcinogenic substance is unknown, it is believed to be particulate in nature, as spouses of these workers are also at increased risk, and areas with exposure greater than 5 mg/m^3^ of this type of dust are harmful to work (27, 28). It is estimated that the woodworkers’ risk of developing the tumor is 500 times greater than that of the unexposed male population and almost 900 times more than that of the general population, with a reported average exposure period of about 40 years (29).

The estimated population of cork workers in the EU is 70.000 (mainly Portugal, Spain, and Italy), constituting a leader in the production, processing and trade of cork. The Iberian Peninsula is responsible for 80.1% of world production. Portugal is the most significant worldwide cork processor (49.6% of global production), with about one thousand companies operating mainly in two districts – Aveiro and Setúbal – mostly having a family level dimension (85% of companies have fewer than 20 employees) (30).

However, there are a few data in the literature linking cork to ITAC: two Italian epidemiological series conducted recently in Italy [Lombardy (18) and Varese (19) regions] documented 1 and 2 cases, respectively. The Lombardy registry of SNC collected all the cases occurring among subjects with residence in the region at the time of the first diagnosis from about 9.6 million people in 2008 to about 9.9 million in 2011 (almost the total population in Portugal). In the period 2008–2001, it was recorded 210 SNC cases, 44 (21%) ITACs, including 1 non-smoking woman exposed to cork dust (18). The other study concerned the experience, between February 2010 and August 2011, of a multidisciplinary postoperative care group at the Otorhinolaryngological Department of the University of Insubria, Varese. The eligible population included 73 patients with SNC, 39 (60%) ITACs, including 2 cases of workers employed in the cork industry (19).

The carcinogenesis of ITAC is still far from being fully explained, some advances have been made in recent years. ITAC seems to develop specifically from the olfactory cleft, suggesting local factors (31). The organic dusts can lead, by perpetuated foreign body reaction, to a chronic inflammation process and, consequently, contribute to the mechanism of tumorigenesis (1, 32). Furthermore, patients with ITAC demonstrate a higher rate of the CYP1A1 codon 461 polymorphism and SDTM1 null genotype compared with the general population, suggesting that genetic susceptibility is necessary for tumor development, thus explaining why most woodworkers are not affected by this cancer (13, 33).

At this point, it is impossible to discriminate the underlying mechanisms for the development of ITAC secondary to prolonged cork exposure. We can speculate, saying that the pathogenesis may be common to that already described for other occupational agents (including wood dust) (1, 31). Other exposures (formaldehyde, smoking habits, etc.) may also be confounding factors, and can be hard to inventory retrospectively. Emission of volatile organic compounds, particularly formaldehyde from cork and wood-based products, can affect the air quality of indoor environments. Thermal treatments, the use of resins and other industrial processes may lead to the appearance of volatile compounds, and thus, increasing the risk of ITAC (34).

Out of curiosity, cork exposure has been previously associated with another disease such as pneumoconiosis (suberosis), described in 1955 by Cancella (35). About two decades later, Pimentel and Avila (36) described in bronchial biopsy specimens a chronic stage of alveolitis with extensive interstitial fibrosis and pronounced infiltration of lymphocytes, histiocytes, and granulomata of sarcoid-like appearance. Cork dust was constantly found in the granular pneumocytes in the alveoli, in the histiocytes of the septal infiltrates, and in the granulomata. The authors appointed two main factors for the lung disease pathogenesis: cork dust within lesions and the potential immunological reaction against a fungus identified on the cork, *Penicillium frequentans*, having confirmed the existence of precipitating antibodies in the patients’ serum. Recently, another Portuguese group identified potential pathogenic fungi assessment through molecular biology in the cork industry. Viegas et al. (37), identified the *Penicillium glabrum* complex in the samples, corroborating the high prevalence of this species in the cork.

It was even identified a fingerprint of cork fungi with various agents present in addition to the genus *Penicillium*, including genus *Aspergillus*, *Chrysonilia*, *Cladosporium*, *Mucor* and *Trichoderma* (38). Establishing associations between dysbiosis and cancer onset and progression as well as patient outcomes constitutes an area of intense research at the precise moment (39, 40).

Thus, in parallel to what has been observed for other occupational agents and in what has already been described in another chronic disease secondary to cork dust, it can be supposed that the ITAC associated with cork dust will have a multifactorial aetiopathogenic origin. It might be caused, among other causes, by a chronic inflammatory process promoted by the agent *per se* or by an immune response to a biological agent (such as a fungus or other local microbiota from cork or the host itself) in an individual with a certain genetic susceptibility.

Given the epidemiological data presented, the rarity of this entity and the fact that most cases of this cancer in central and southern Portugal are treated at our institution, we decided that it would be relevant to determine the frequency, in this cohort, of the possible history of a professional activity that would deal directly with cork. It is noteworthy that about half of patients report a history of professional exposure to cork. Despite the small numbers, it is interesting to know that in recent years the incidence of cases in our center has been decreasing. Does this reflect chance, referral to other centers or the reflection of protective measures that have been implemented in the medium term.

Although this retrospective series does not allow us to calculate association measures between prolonged cork exposure and ITAC, these findings may lead to further investigation to test this potential link. The awareness of our data underlines the importance of considering occupational risks such as cork exposure and may lead to reinforcement of preventive and protective measures and politics.

The current analysis is limited by several factors, including its retrospective nature, the single institution source of data, the re-staging according to the TNM latest edition for data with a time indentation over 30 years, the fact that not all medical records are uniformly and adequately filled, the inability to obtain additional information regarding patients lost during follow-up, including the type of contact with the cork and its protective equipment.

Nevertheless, the authors believe that the current findings represent a piece of relevant epidemiological information.

## Conclusion

Intestinal-type adenocarcinoma is an uncommon malignancy. We confirmed a tendency to late diagnosis and occupational exposure relation as a predisposing factor. An accurate and rapid histological evaluation allows for an early staging and is the key for planning the combined modality therapy.

In our cohort, we observed a high prevalence of cork occupational exposure in ITAC. This finding may lead to the implementation of protection measures and suggest a potential link to be further studied.

## Data Availability Statement

The raw data supporting the conclusions of this article will be made available by the authors, without undue reservation.

## Ethics Statement

This study was approved by the Research Ethics Committee of the IPOL-FG, under protocol number UIC_989.

## Author Contributions

DAC, AMn, TAn, and AMr: conception and design. DAC, AMn, TAn, SE, and AMr: development of methodology. DAC, AMn, TAn, SE, AC, and AMr: acquisition, analysis, and interpretation of the data, and writing, review, and/or revision of the manuscript. IS, MF, TAl, AC, JF, and AMr: manuscript supervision. All authors have given their permission for publishing the manuscript, have read the submission and agreed to be listed as co-authors.

## Conflict of Interest

The authors declare that the research was conducted in the absence of any commercial or financial relationships that could be construed as a potential conflict of interest.

## Footnotes

1 www.r-project.org/
